# Photothermal MOF–Alginate Hydrogel Beads for Solar-Driven Atmospheric Water Harvesting

**DOI:** 10.3390/gels12100869

**Published:** 2026-09-25

**Authors:** Roberto Di Pietro, Vincenza Brancato, Andrea Frazzica, Alessandro Sinopoli, Elpida Piperopoulos

**Affiliations:** 1 Consiglio Nazionale delle Ricerche, Istituto di Tecnologie Avanzate per l’Energia “Nicola Giordano” (CNR ITAE), Via S. Lucia sopra Contesse, 5, 98126 Messina, Italy; robertodipietro1@cnr.it (R.D.P.); vincenza.brancato@cnr.it (V.B.); andrea.frazzica@cnr.it (A.F.); elpida.piperopoulos@unime.it (E.P.); 2 Department of Chemical, Biological, Pharmaceutical and Environmental Sciences, University of Messina, V.le F. Stagno d’Alcontres 31, 98166 Messina, Italy; 3 Department of Engineering, University of Messina, C.da Di Dio, Vill. S. Agata, 98166 Messina, Italy

**Keywords:** atmospheric water harvesting, alginate hydrogel, MOF–hydrogel composite, photothermal regeneration, graphene oxide, stimuli-responsive gels, MIL-101(Cr), MIL-100(Fe)

## Abstract

Atmospheric water harvesting (AWH) has emerged as a promising strategy for decentralised freshwater production in regions where conventional water infrastructure is limited. Among the various approaches, sorption-based systems employing metal–organic frameworks (MOFs) are particularly attractive due to their high surface area and tunable pore chemistry. In this work, we report the development of structured photothermal composite beads based on Ca^2+^-crosslinked sodium alginate (SA) incorporating MIL-101(Cr) and MIL-100(Fe) MOFs together with graphene oxide (GO) as a solar absorber. The resulting MOF–alginate–GO beads combine high water adsorption capacity with efficient photothermal regeneration and good mechanical robustness. The Ca^2+^-crosslinked alginate network acts as a water-permeable hydrogel scaffold, enabling repeated swelling–drying cycles while integrating MOF sorption capacity and GO-mediated light-to-heat conversion. Dynamic vapour sorption measurements reveal characteristic cooperative adsorption behaviour, with MIL-101(Cr) beads exhibiting an earlier adsorption step at ~30–40% RH, while MIL-100(Fe) beads activate at ~45–55% RH. Under simulated solar irradiation (1 sun), the incorporation of GO enhances solar-to-thermal conversion, increasing the fraction of desorbed water by approximately 22%, 12%, and 8% relative to the corresponding GO-free SA, Fe/SA, and Cr/SA formulations, respectively. These results highlight the potential of MOF–alginate photothermal composites as scalable sorbents for solar-driven atmospheric water harvesting, particularly in moderate-humidity environments.

## 1. Introduction

Freshwater scarcity is intensifying worldwide due to population growth, climate change, and increasing stress on conventional water resources. Atmospheric water harvesting has therefore emerged as a promising approach for decentralised freshwater production, particularly in arid and remote regions where conventional desalination or distribution infrastructure is costly or unavailable [1]. Although the atmosphere contains a vast and continuously replenished reservoir of water vapour, its low density and spatial dispersion require efficient sorbents and energy-conscious regeneration strategies for practical extraction. Among existing AWH technologies, sorption-based systems have attracted particular attention because they can capture moisture passively, often during cooler nighttime conditions, and release water using low-grade heat sources such as sunlight, enabling off-grid operation with minimal energy input [2,3,4,5]. However, translating high laboratory adsorption capacity into deployable devices requires sorbents that simultaneously combine high working capacity, low regeneration temperature, solar compatibility, scalability, and long-term operational stability.

Metal–organic frameworks have attracted extensive interest as next-generation AWH sorbents owing to their exceptionally high surface area, tunable pore chemistry, and ability to reversibly adsorb water at low to moderate relative humidity (RH) [3,6,7]. Hydrolytically stable frameworks such as MOF-801 and MOF-303 enabled some of the earliest demonstrations of practical sorption-based AWH devices, highlighting the potential of MOF materials for sunlight-driven water production. Device studies based on these materials have reported water productivities ranging from ~0.1 to 1.0 L·kg·MOF^−1^·day^−1^ depending on humidity conditions, cycle duration, and system configuration [2,7]. In addition to these arid-climate systems, frameworks belonging to the MIL-100/MIL-101 family have attracted increasing attention because their large cages and hydrophilic pore windows enable cooperative water adsorption and high working capacities at moderate humidity levels, making them attractive candidates for environments with RH above ~40–50%.

Despite their promising adsorption properties, MOF powders present several limitations for practical deployment in atmospheric water harvesting devices, including poor solar absorption, limited thermal conductivity, pressure drop in packed beds, and handling challenges during repeated adsorption–desorption cycles [2,8]. These constraints have stimulated increasing interest in structured and composite MOF systems that incorporate additional functionalities beyond adsorption alone. In particular, integrating photothermal components into MOF-based sorbents has emerged as an effective strategy to overcome the intrinsically weak solar–thermal conversion of many MOFs, enabling faster sunlight-driven regeneration and higher operational cycle frequency [9,10]. Early solar-driven MOF devices relied primarily on direct solar heating and were therefore strongly limited by heat management and absorber efficiency; for example, a widely cited MOF-801 device concept produced approximately 0.2 L·kg_MOF_^−1^·day^−1^ at 20% RH [11]. To improve solar utilisation, broadband absorbers such as carbon black, graphite, and carbon nanotubes have been incorporated into MOF materials, enhancing optical absorption and thermal conductivity without altering the intrinsic water-sorption chemistry of the framework. For instance, a MOF-801/CNT composite exposed to 1 kW·m^−2^ simulated sunlight released more than 90% of its stored water within 10 min and completed desorption in about 15 min, with the sorbent temperature rapidly reaching ~96 °C [12]. More broadly, highly absorbing carbonaceous photothermal phases, including MOF-derived porous carbons, have demonstrated strong sunlight utilisation (up to ~97% absorption) and enhanced water production rates, reaching about 380 mg·g^−1^·h^−1^ at 40% RH and up to 1.77 L·kg^−1^ collected during daylight hours under 25 °C and 40% RH conditions [13]. Recent MXene/MOF/SA monoliths operating in a dual-layer configuration achieved water productivities of ~57.8 mL H_2_O kg_MOF_^−1^·h^−1^ at 20% RH (25 °C), with simulated sunlight heating producing temperature increases of up to ~40% [14]. These demonstrations highlight the potential of combining MOF sorption with photothermal materials to enhance solar-driven AWH performance.

In addition to composition, sorbent morphology plays a critical role in the scalability and practical performance of AWH systems. Transitioning from powders to structured forms such as pellets, monoliths, aerogels, and beads improves mechanical stability, facilitates handling, and enables better control of mass and heat transfer in adsorption beds [8,15,16]. From a system perspective, structuring is also essential to mitigate packed-bed pressure drop, avoid dusting, and maintain permeability at practical airflow rates, constraints repeatedly identified as major barriers when translating MOF powders into real devices [17]. Moreover, dense packing and hardware constraints can prevent powder beds from reaching full saturation and desorption due to increased mass-transfer resistance in practical modules [18]. Alginate-based bead architectures are particularly attractive due to their low cost, renewability, and ability to form mechanically robust hydrogels through ionic crosslinking with divalent cations such as Ca^2+^ [17,19,20]. The resulting bead structures combine structural integrity with good vapour permeability and can be easily packed into adsorption columns while maintaining low pressure drop. In MOF–alginate hybrid beads, the hydrogel matrix facilitates vapour diffusion and water retention while providing mechanical integrity during repeated adsorption–desorption cycling [17,21]. Consequently, bead-type MOF–hydrogel composites are increasingly considered a practical pathway toward scalable AWH modules.

While several studies have explored MOF–polymer composites or photothermal AWH sorbents, many reported systems rely either on salt-assisted hygroscopic adsorption, highly humid operating conditions, or complex monolithic architectures that may limit scalability and practical handling. In addition, photothermal AWH demonstrations often emphasise heating performance without directly correlating adsorption behaviour, structured morphology, and sunlight-driven regeneration in a single sorbent architecture. Consequently, there remains a need for MOF-based materials without additional hygroscopic-salt loading that combine reversible adsorption at moderate humidity with efficient solar-driven regeneration in scalable and mechanically robust forms. Recent reports on MOF–alginate beads and salt-assisted hydrogel sorbents have demonstrated high water uptake under humid conditions; however, the integration of MOF-driven adsorption with GO-assisted solar photothermal regeneration in a simple bead architecture, without additional hygroscopic-salt impregnation, remains comparatively less explored.

Here, we report Ca^2+^-crosslinked sodium alginate beads embedding MIL-101(Cr) and the Fe-based framework MIL-100(Fe), augmented with graphene oxide (GO) as a photothermal component. In this light-responsive MOF–hydrogel bead architecture, the MOF provides selective and reversible water adsorption, GO enhances solar-to-thermal conversion to accelerate regeneration, and the alginate matrix ensures mechanical robustness and processability. The adsorption performance, solar-driven desorption behaviour, and cyclic stability of the composite beads are investigated under controlled conditions to evaluate their potential for practical atmospheric water harvesting applications.

## 2. Results and Discussion

### 2.1. Design Rationale and Fabrication of MOF–Alginate Beads

The composite sorbents were designed to combine the high water adsorption capacity of MOFs with the structural robustness of a polymeric matrix and the photothermal functionality required for solar-driven regeneration. In this architecture, MIL-101(Cr) and MIL-100(Fe) act as the primary adsorption phases, graphene oxide (GO) functions as a broadband photothermal absorber, and Ca^2+^-crosslinked sodium alginate provides a mechanically stable hydrogel framework that enables the formation of millimetre-scale beads. SEM and XRD characterisation of the pristine MIL-101(Cr) and MIL-100(Fe) used for bead fabrication are provided in Figures S1 and S2. This hierarchical design aims to overcome several limitations of MOF powders, including poor handling properties, limited solar absorption, and high pressure drop when used in packed beds. In this formulation, Ca^2+^ is the intentional crosslinking ion responsible for formation of the alginate hydrogel network. Cr and Fe are present as framework metal nodes within the preformed MIL-101(Cr) and MIL-100(Fe) particles, respectively, and are not introduced as free alginate-crosslinking ions. Therefore, although interfacial interactions between alginate carboxylate groups and exposed MOF surface sites may occur, the different sorption behaviours of the Cr- and Fe-containing beads are attributed primarily to the distinct pore structures, pore volumes, and water-filling steps of the embedded MOF phases rather than to deliberate Cr- or Fe-mediated crosslinking of alginate.

A GO loading of 10 wt.% with respect to the MOF content was selected based on prior reports on photothermally active MOF–polymer composites, where a similar mass fraction of light-absorbing additives was found to be sufficient to achieve efficient in situ heat generation without compromising the adsorption matrix [15,17]. This composition was selected to combine water sorption and photothermal functionality; the GO loading was not systematically optimised in this study. The hydrophilic functional groups of alginate (–OH and –COO^−^), together with the internal porosity of MOFs, provide abundant active sites for water adsorption through hydrogen bonding and capillary condensation. GO may contribute additional oxygenated surface sites, but its primary role in these composites is photothermal rather than adsorptive.

The water capture in the composite beads proceeds through a combination of physical adsorption within MOF micropores and alginate free volume, as well as bound hydration around Ca^2+^ crosslinking sites and surface functional groups. Upon solar exposure, the GO phase rapidly converts absorbed light into heat, accelerating the desorption of bound and confined water molecules and enabling fast water release from the bead interior. The hierarchical integration of alginate chains, MOF crystallites, and GO is expected to favour vapour accessibility during adsorption and local photothermal heating during regeneration. The SEM, DVS, and solar-heating results are consistent with this interpretation, although direct transport and thermal-conductivity measurements were not performed.

### 2.2. Characterisation of Pristine MOFs and Composite Beads

#### 2.2.1. Phase Purity of Pristine MOF Powders by PXRD

Powder X-ray diffraction (PXRD) was used to verify the crystallinity and phase purity of the pristine MOF powders prior to their incorporation into the alginate beads. The MIL-101(Cr) diffraction pattern presented in Figure S1 shows the characteristic reflections reported for the chromium terephthalate framework originally described by Férey et al., including the intense low-angle peaks that arise from its large cubic unit cell. No additional reflections attributable to chromium oxides or other secondary phases were detected, indicating phase-pure synthesis and good crystallinity [22,23]. The absence of the grimaldiite reflection at $2\theta \approx 20^{\circ }$ further supports the phase purity of MIL-101(Cr), excluding common chromium oxyhydroxide impurities.

The MIL-100(Fe) sample similarly displays the characteristic low-angle reflections of the Fe-BTC framework, with peaks at 2θ ≈ 3.4°, 4.0°, 4.8°, 5.3°, 5.9°, and 6.3°, corresponding to the (220), (311), (400), (331), (422), and (333) planes of the cubic structure. The clear visibility of these low-angle reflections and their narrow full width at half maximum indicate well-developed crystalline domains and long-range structural order. Importantly, no diffraction peaks corresponding to iron oxides or amorphous Fe-BTC phases were observed, confirming the successful formation of phase-pure MIL-100(Fe) [24,25]. These results demonstrate that the synthetic procedures adopted here yield highly crystalline MOF powders suitable for incorporation into the composite beads.

To verify whether the crystalline MOF structure was retained after bead fabrication and subsequent cycling, PXRD patterns were also collected for the fresh and cycled Cr/SA/GO and Fe/SA/GO composites and compared with those of the corresponding pristine MOF powders (Figure S1). In both systems, the main diffraction features characteristic of MIL-101(Cr) and MIL-100(Fe) remain detectable after incorporation into the alginate/GO matrix, indicating that a crystalline MOF component remains present after bead formation. Compared with the pristine powders, the fresh composite beads show lower relative peak intensities, peak broadening, and a more pronounced background contribution. These changes are expected for a diluted crystalline phase embedded within an amorphous polymeric matrix and may additionally reflect differences in particle orientation and scattering geometry. Importantly, the characteristic low-angle reflections of the corresponding MOFs remain evident, indicating that bead fabrication does not induce complete loss of framework crystallinity. The successful formation of the composites was also assessed by SEM, FTIR spectroscopy, and water-vapour sorption measurements. In particular, the retention of MOF-related FTIR bands and framework-specific DVS adsorption profiles confirms that the characteristic chemical and sorption features of MIL-101(Cr) and MIL-100(Fe) are preserved after incorporation into the Ca^2+^-crosslinked alginate matrix.

The textural properties of the pristine MOF powders are summarised in Table S1, confirming the highly porous character of both frameworks and supporting the different water-sorption capacities observed for the Cr- and Fe-based composites.

#### 2.2.2. Chemical Fingerprints of MOF–Alginate Beads by FTIR

Fourier-transform infrared spectroscopy (FTIR) was used to further verify the chemical composition of the MOF–alginate composites and to assess whether the characteristic functional groups of the individual components were retained after bead formation. All composite spectra display a broad absorption in the 3400–3200 cm^−1^ region, assigned to O–H stretching from hydrated alginate, adsorbed water and oxygenated surface groups, together with weaker C–H stretching contributions around 2920–2850 cm^−1^ (Figure 1). The intense bands observed in the 1600–1590 cm^−1^ and 1410–1370 cm^−1^ regions are attributed to the asymmetric and symmetric stretching modes of carboxylate groups, respectively. These features arise from both the alginate matrix and the coordinated carboxylate linkers of the MIL frameworks and are consistent with Ca^2+^-mediated crosslinking of the alginate network and preservation of the MOF carboxylate coordination environment. Additional bands in the 1300–1000 cm^−1^ range are assigned to C–O, C–O–C, and C–OH stretching modes associated with the polysaccharide backbone of alginate and oxygenated functionalities of GO, while bands below 900 cm^−1^ are related to aromatic linker vibrations and metal–oxygen framework modes. In the MIL-101(Cr)-based beads, bands at approximately 1595–1590, 1397, 1290, 1024–1020, 880, 815–740, and 560–500 cm^−1^ confirm the retention of the chromium terephthalate framework within the alginate matrix. Similarly, the MIL-100(Fe)-based beads retain the main spectral features at approximately 1600, 1370, 1020–1000, 760–700, and 620 cm^−1^, consistent with the presence of the Fe-trimesate structure (also visible in Figure S5). The GO-containing samples show the same principal MOF/alginate bands, with additional contributions in the C–O/O–H regions, indicating successful incorporation of the photothermal additive without disrupting the main framework fingerprints.

The comparatively intense O–H stretching envelope observed for Fe/SA may indicate a larger contribution from adsorbed or bound water and hydrogen-bonded hydroxyl groups within the MIL-100(Fe)/alginate composite. However, this feature should be interpreted cautiously because ATR-FTIR band intensities depend on sample contact, residual hydration and spectral normalisation. In addition, Fe is present primarily as framework metal nodes within MIL-100(Fe), whereas Ca^2+^ is the ion responsible for alginate crosslinking; therefore, the Fe/SA spectrum should not be interpreted as evidence of intentional Fe^3+^-mediated alginate crosslinking.

Overall, the FTIR spectra support the formation of MOF–alginate–GO composite beads and confirm that the characteristic chemical functionalities of the MOF frameworks and alginate matrix are preserved after processing.

#### 2.2.3. Morphology of the Composite Beads by SEM

Scanning electron microscopy (SEM) further elucidates the morphology of the synthesised materials. MIL-100(Fe) crystals display the faceted octahedral morphology typically associated with this framework (see Figure S2), with particle sizes generally below 1 µm and moderate aggregation [24,26]. In contrast, MIL-101(Cr) exhibits polyhedral particles with relatively smooth surfaces (see Figure S2), consistent with hydrothermal synthesis routes reported in the literature. These morphological characteristics are consistent with the high crystallinity observed in the XRD patterns [22].

After incorporation into the alginate matrix, the resulting composite beads exhibit a nearly spherical morphology with diameters of approximately 1–2 mm, typical of drop-gelation processes. SEM images depicted in Figure 2 reveal that the reference SA/GO beads possess a relatively smooth and compact external surface, reflecting the densification of the alginate matrix during drying. The outer layer appears as a continuous polymer “skin” with only minor micro-wrinkling, suggesting that the hydrogel collapses partially as water is removed.

The presence of MOF particles significantly modifies this morphology. In Cr/SA/GO beads, the outer surface becomes noticeably rougher and more textured, displaying micron-scale ridges and shallow pits distributed across the bead surface. This reticulated structure likely arises because the rigid MOF crystallites disrupt the collapse of the polymer network during drying, thereby preserving additional macroporosity. Such structural features are advantageous for vapour transport because they create additional pathways for mass transfer between the external environment and the interior of the bead.

Fe/SA/GO beads show a somewhat different morphology. At higher magnification, the surface appears as a finer mesh-like microstructure with fewer large protrusions, indicating good dispersion of the MOF particles within the polymer matrix rather than the formation of large agglomerates. Importantly, the beads remain mechanically intact and free of cracks, demonstrating that the Ca^2+^-crosslinked alginate network provides sufficient cohesion to accommodate the embedded inorganic phases.

SEM–EDS elemental mapping was further used to assess the spatial distribution of the inorganic components within the analysed bead surface regions (Figure S3). In both Fe/SA/GO and Cr/SA/GO beads, Ca is distributed across the mapped area, consistent with Ca^2+^-crosslinking of the alginate matrix. The Fe map of Fe/SA/GO and the Cr map of Cr/SA/GO show that the corresponding MOF-derived metal signals are detected throughout the analysed regions, supporting successful incorporation and spatial distribution of MIL-100(Fe) and MIL-101(Cr) within the alginate/GO beads; however, full three-dimensional homogeneity throughout the bead interior was not assessed because cross-sectional elemental mapping was not performed. These maps complement the FTIR and PXRD results by showing the spatial distribution of Ca and the corresponding MOF-derived metal signals within the analysed surface regions.

Cross-sectional SEM images of the Fe/SA/GO and Cr/SA/GO beads are provided in Figure S4, showing that the composite matrix extends throughout the bead interior; the larger internal cavities visible in some regions are attributed to entrapped air bubbles formed during bead preparation rather than to intrinsic material porosity.

Overall, SEM shows that the reference SA/GO bead forms a dense outer shell, whereas MOF-loaded beads develop a rougher, more open surface. The absence of micron-sized MOF clusters and the presence of a rougher external surface in the Cr- and Fe-containing beads are consistent with the higher equilibrium uptakes and with the higher released-water fractions after 90 min of solar irradiation observed for the composites.

### 2.3. Water-Vapour Sorption Behaviour

Water-vapour adsorption–desorption isotherm measurements of the composite beads were conducted at 30 °C over a relative humidity range of 5–90%. The resulting isotherms, reported in Figure 3, exhibit the characteristic sigmoidal profiles typically associated with cooperative water adsorption in porous materials.

The water-vapour adsorption–desorption isotherms of the Ca–alginate/GO beads loaded with MIL-101(Cr) and MIL-100(Fe) show the hallmarks of type-V behaviour: an S-shaped adsorption branch followed by a pronounced H3/H4-like hysteresis loop. The initial, gently rising region at low relative humidity reflects adsorption on polar sites of the alginate/GO matrix together with the most accessible sites of the embedded MOFs. A sharp increase in uptake then appears, the cooperative-filling “step”, whose position fingerprints the framework. Beads containing MIL-101(Cr) display an earlier composite-level adsorption step (≈30–40% RH), whereas MIL-100(Fe)-containing beads show their main transition at higher humidity (≈45–55% RH). At high RH both bead types continue to gain mass, reaching substantial total capacities; at a fixed RH near saturation, the MIL-101(Cr) beads achieve the highest loadings, with MIL-100(Fe) beads slightly lower and the polymeric bead without MOF the lowest.

The desorption branches lag behind adsorption, producing open loops whose breadth is governed less by the intrinsic MOF cages and more by transport through the polymer network. Capillary condensation within interparticle voids and meso/macropores of the bead, together with humidity-induced swelling of alginate, slows vapour egress and leads to kinetic trapping during drying. Introducing MOF crystallites stiffens the composite and provides rigid microporous pathways, which narrows the hysteresis relative to the MOF-free bead, yet some irreversibility remains because water must still percolate through the polymer–GO scaffold. This behaviour has practical consequences. In mid-RH conditions typical of many indoor or arid–semiarid evening climates (30–60% RH), MIL-101(Cr) beads are advantageous because their step lies squarely in this window, maximising uptake per cycle. Under persistently humid conditions (>60% RH), MIL-100(Fe) beads approach the performance of MIL-101(Cr) as their step is fully engaged. Regeneration is expected to be faster and less energy-intensive for the MOF-rich beads, which exhibit smaller hysteresis loops and lower residual loading at moderate RH on the desorption branch.

For comparison, SA-only beads (no MOF) show the smallest overall uptake and lack a pronounced cooperative step, indicating that sorption is dominated by polymer hydration rather than cage filling. Their hysteresis loop is widest, consistent with matrix swelling, capillary condensation in macropores, and slow back-diffusion during drying, which leaves a higher residual loading at moderate RH.

The pristine MOF powders exhibit distinct water-vapour adsorption–desorption profiles at 30 °C (Figure S6). MIL-100(Fe) shows a progressive, multistep increase in water uptake: an initial rise occurs between approximately 20 and 30% RH, followed by a further increase between 40 and 50% RH. Above this region, uptake increases more gradually, reaching approximately 0.53 g·g^−1^ at 70% RH. In contrast, MIL-101(Cr) exhibits comparatively limited uptake below approximately 40% RH, followed by a pronounced adsorption step between 40 and 60% RH and a terminal loading of approximately 0.95 g·g^−1^ at 70% RH. Thus, the higher water capacity of the pristine Cr framework is associated with a larger uptake following the main filling transition, rather than an earlier adsorption onset relative to MIL-100(Fe). The higher terminal capacity is consistent with the larger pore volume reported for MIL-101(Cr) in Table S1, although pore volume alone does not determine the position or shape of the water-adsorption step.

Incorporation into the Ca^2+^-crosslinked alginate matrix modifies these powder sorption profiles. The Cr-containing beads exhibit a broader adsorption transition than the pristine MIL-101(Cr) powder, with appreciable uptake developing before the sharp filling step observed for the powder. The Fe-containing beads likewise show a modified transition while retaining lower high-RH uptake than the Cr-containing formulations. These differences are consistent with the combined contributions of MOF adsorption and hydration of the alginate-based matrix, together with possible changes in pore accessibility after bead formation. Consequently, the composite isotherms should not be interpreted as simple scaled-down replicas of the powder isotherms, and their adsorption-step positions should be evaluated separately. Comparisons of uptake must also be made at the same RH and on a consistent mass basis, since the powder measurements extend to 70% RH, whereas the bead isotherms extend to 90% RH. In addition, uptake per gram of bead includes water associated with both the MOF and the polymer-containing matrix.

Both pristine powders exhibit adsorption–desorption hysteresis around their principal filling regions. Hysteresis is also evident in the composite beads, where retention of water within the MOF pores may be accompanied by hydration and swelling of the alginate matrix and transport limitations under the applied DVS protocol. These contributions may modify the separation between adsorption and desorption branches, but their individual roles were not independently quantified. The combined powder and bead measurements therefore show that the higher high-RH uptake of the Cr-containing formulations is consistent with the greater water capacity of the parent MIL-101(Cr), while bead formation modifies the humidity-dependent sorption response. These observations support the contribution of the embedded MOFs to water uptake without establishing unchanged pore accessibility or a direct correspondence between the adsorption steps of the powders and composites.

Overall, the composites retain substantial water-sorption capacity, while their humidity-dependent uptake and hysteresis reflect the combined influence of the embedded MOF and the alginate-based matrix. This validates the beads as function-retaining MOF carriers and explains their strong storage yet slower, more irreversible drying relative to the free MOF powders. The isotherms indicate that both composites are well suited to cyclic water capture, with MIL-101(Cr) beads favouring lower-humidity operation and MIL-100(Fe) beads performing robustly at higher humidity; the magnitude and shape of the hysteresis underscore the central role of the alginate/GO matrix in dictating mass-transport and desorption kinetics. The water uptake mechanism in the composite beads should therefore be interpreted as a combination of physical adsorption, cooperative pore filling and hydrogel-assisted water retention, rather than as simple monolayer adsorption. At low RH, water molecules interact with polar sites of the Ca^2+^-crosslinked alginate matrix, oxygenated GO functionalities and accessible hydrophilic sites of the embedded MOF particles through hydrogen bonding and physisorption. As RH increases, cooperative water clustering and pore filling occur within the MIL cages, giving rise to the characteristic S-shaped adsorption steps observed for the MOF-containing beads. At higher RH, additional contributions from alginate hydration, swelling, interparticle voids, and capillary condensation become increasingly relevant, as reflected by the hysteresis between adsorption and desorption branches. Quantitative adsorption enthalpies were not determined in the present work because this would require temperature-dependent water-vapour isotherms or calorimetric measurements; therefore, no specific ΔH_sorp_ values are claimed.

From an application perspective, these sorption characteristics suggest that MIL-101(Cr) beads may be particularly advantageous in environments with moderate humidity levels (approximately 30–60% RH), where the adsorption step falls within the operating humidity window. MIL-100(Fe) beads, by contrast, may perform better under consistently higher humidity conditions where their cooperative adsorption step becomes fully activated.

To complement the equilibrium sorption analysis, time-resolved adsorption–desorption measurements were performed for the two GO-containing MOF composites at 10, 40, and 70% RH, followed by desorption under dry conditions at 80 °C (Figure 4). These measurements provide information on the rate at which the beads approach their humidity-dependent water loading and subsequently release the adsorbed water under dry conditions.

The kinetic measurements show the same qualitative increase in water uptake with RH as the stepped DVS isotherms. However, the two datasets were acquired using different experimental protocols, separate fixed-RH adsorption measurements, and sequential RH steps, respectively, and their terminal uptake values should not be treated as directly equivalent. Cr/SA/GO reached terminal mass gains of approximately 6.8, 25.7, and 47.1% at 10, 40, and 70% RH, respectively, whereas the corresponding values for Fe/SA/GO were approximately 6.0, 22.0, and 35.5%. The difference between the two composites became particularly evident at 70% RH, where Cr/SA/GO combined a higher final uptake with a more rapid approach to the high-loading region. At 40% RH, the initial uptake behaviour of the two composites was more comparable, although Cr/SA/GO subsequently reached a higher terminal loading. Upon switching to dry conditions, both composites exhibited a rapid initial mass loss followed by a slower residual desorption tail. Cr/SA/GO showed a markedly faster return towards the dry baseline over the investigated humidity range, whereas Fe/SA/GO displayed a more persistent desorption tail. The difference was particularly evident after adsorption at low and intermediate RH, indicating slower removal of the remaining adsorbed water from the Fe-containing composite.

The different kinetic responses of the two composites can be rationalised in terms of the combined framework chemistry and pore architecture of the embedded MOFs. MIL-101(Cr) possesses a substantially larger BET surface area and pore volume than MIL-100(Fe) in the present study, together with larger cages and wider pore apertures. Water adsorption in these frameworks involves initial interaction with hydrophilic framework sites followed by cooperative water-cluster growth and progressive pore filling as the relative humidity increases. The larger accessible pore volume of MIL-101(Cr) therefore provides a greater capacity for water uptake, while its wider pore windows may also facilitate water-vapour exchange during both adsorption and desorption. This interpretation is consistent with the higher terminal uptake and faster overall water exchange observed for Cr/SA/GO, particularly at 70% RH. Conversely, the lower capacity and more persistent desorption tail of Fe/SA/GO are compatible with the smaller pore volume and narrower pore apertures of MIL-100(Fe).

Cr/SA/GO reached higher terminal water loadings than Fe/SA/GO at all three investigated RH values. The clearest difference in adsorption behaviour was observed at 70% RH, where Cr/SA/GO approached its terminal loading more rapidly. These humidity-controlled DVS measurements should, however, be regarded as complementary to rather than representative of the solar-driven regeneration experiments discussed in the following section.

### 2.4. Solar-Driven Photothermal Regeneration

The impact of photothermal heating on water release was evaluated by measuring the fraction of adsorbed water desorbed during illumination. Prior to these experiments, the samples were equilibrated for 18 h at 70% RH to establish a reproducible hydrated state.

Under simulated 1-sun irradiation, all bead formulations exhibited a pronounced increase in surface temperature during the first 10 min, reaching values between 42.9 and 52.1 °C (Figure 5). Subsequent temperature changes were smaller, with surface temperatures ranging from 48.5 °C for SA to 56.8 °C for Fe/SA/GO after 90 min. GO-containing formulations exhibited higher surface temperatures than their corresponding GO-free controls. At 90 min, the temperatures were 48.5 and 54.3 °C for SA and SA/GO, 53.6 and 56.8 °C for Fe/SA and Fe/SA/GO, and 51.0 and 53.1 °C for Cr/SA and Cr/SA/GO, respectively. These observations support the contribution of GO to photothermal heating under the tested conditions.

Representative FLIR thermal blending images acquired after 90 min of irradiation are provided in Figure S7 as qualitative visual support for the measured surface-temperature response of the different bead formulations.

The mass of all equilibrated beads was recorded before the desorption experiment and 90 min after the irradiation started to determine the fraction of water released (Figure 6). After 90 min of irradiation, GO increased the fraction of initially adsorbed water released by approximately 22%, 12%, and 8% relative to the corresponding GO-free SA, Fe/SA, and Cr/SA formulations, respectively, confirming GO’s role as a local photothermal booster. Absolute water uptake (blue bars, right axis) is highest for the MOF beads, so pairing MOF storage with GO heating maximises the released water per cycle: Fe/SA/GO and Cr/SA/GO combine high capacity with improved regeneration. SA shows the lowest released-water fraction under the tested irradiation conditions.

Taken together, the data show a clear synergy: the MOFs supply high equilibrium loading, while GO supplies fast, localised heating that tips the desorption equilibrium and increases the fraction of water released after 90 min under sunlight. Fe/SA/GO reached the highest surface temperature, whereas Cr/SA/GO showed the highest released-water fraction after 90 min.

### 2.5. Cycling Performance and Durability

#### 2.5.1. Short-Term Wetting–Drying Repeatability

The cyclic water uptake of the beads was first evaluated through a short-term gravimetric wetting–drying test under controlled laboratory conditions. In this experiment, the wetting step was performed in an environmental chamber at 35 °C and 90% RH for 2 h, while regeneration was carried out in an oven at 80 °C for 12 h. This oven-regeneration step was not intended to simulate direct solar heating under natural illumination. Instead, it was used to impose a reproducible and complete drying condition, allowing the post-drying mass and water uptake to be compared consistently over consecutive cycles. The solar-driven regeneration behaviour of the beads was evaluated separately under 1-sun irradiation in Section 2.4.

The tare mass of the empty sample holder was subtracted from all measurements, and the gravimetric water uptake was calculated as

(1) $$q=\frac{m_{wet}-m_{dry}}{m_{dry}-m_{tare}}$$

where $m_{tare}$ is the mass of the empty container, $m_{dry}$ is the total mass after oven drying, and $m_{wet}$ is the total mass after exposure to humid air.

As shown in Figure 7 (left), all bead formulations retained substantial water uptake over ten consecutive wetting–drying cycles. The Cr-containing samples showed higher uptake than the Fe-based analogues, in agreement with the larger pore volume of MIL-101(Cr) and its earlier cooperative filling behaviour observed by DVS. The average uptake shown in Figure 7 (right) followed the order: Cr/SA > Cr/SA/GO > Fe/SA > Fe/SA/GO, with mean values of 0.385 ± 0.034 g·g^−1^ for Cr/SA, 0.367 ± 0.032 g·g^−1^ for Cr/SA/GO, 0.268 ± 0.013 g·g^−1^ for Fe/SA, and 0.241 ± 0.017 g·g^−1^ for Fe/SA/GO. These values confirm that the MOF-loaded alginate beads maintain a meaningful working capacity under repeated humidification and thermal regeneration.

A slight decrease in uptake was observed over the first cycles, particularly for the Cr-containing formulations. Cr/SA decreased from 0.418 to 0.357 g·g^−1^, while Cr/SA/GO decreased from 0.417 to 0.340 g·g^−1^ over ten cycles. The Fe-based samples were more stable, with Fe/SA decreasing from 0.278 to 0.261 g·g^−1^ and Fe/SA/GO remaining nearly unchanged from 0.248 to 0.245 g·g^−1^. This behaviour is more consistent with an initial conditioning of the Ca^2+^-crosslinked alginate/MOF network than with material degradation. Repeated swelling during humidification and contraction during oven drying may reorganise the polymer matrix, remove weakly retained water, or slightly modify accessible free volume, leading to a lower but more reproducible working uptake after the first cycles.

PXRD patterns collected after ten wetting–drying cycles show that characteristic reflections of MIL-101(Cr) and MIL-100(Fe) remain detectable in the corresponding composite beads (Figure S1), although changes in peak intensity and broadening are observed relative to the fresh composites. These results support the persistence of a crystalline MOF component after short-term cycling.

#### 2.5.2. Accelerated Ageing Under Harsh Cycling Conditions

The short-term wetting–drying cycling test should be distinguished from the harsher accelerated ageing experiment performed in the custom cycling apparatus. In the short-term test, wetting and drying were carried out over relatively long periods using an environmental chamber and an oven, allowing the samples to approach well-defined hydrated and regenerated states. By contrast, the accelerated ageing protocol intentionally imposes much more severe conditions, with rapid alternation between adsorption and desorption steps in a confined setup. Adsorption was performed at 30 °C for 10 min, followed by desorption at 80 °C for 7 min. This rapid alternation between humid adsorption and high-temperature drying generates substantially harsher conditions than those encountered in realistic day–night AWH operation, effectively functioning as a stress-testing protocol to probe mechanical integrity and sorption stability.

Comparison of the approximate uptakes at 90% RH in Figure 3 and Figure 8 indicates that Cr/SA/GO and Fe/SA/GO retain approximately 60% and 61% of their initial adsorption capacity, respectively, after more than 2000 accelerated cycles, whereas the uptake of SA/GO remains essentially unchanged. These graphical estimates indicate an approximately 40% reduction in high-RH adsorption capacity for the MOF-containing beads, despite retention of water-sorption functionality. The post-cycling profiles indicate that the basic adsorption function is retained, although the changes in uptake and hysteresis suggest partial restructuring of the alginate matrix and/or altered accessibility of the embedded MOF pores under the harsh rapid-cycling protocol. Therefore, this test should be interpreted as evidence of retained functionality under severe ageing conditions, rather than full preservation of the initial sorption performance.

SEM analysis of the cycled samples, reported in Figure 9, shows that the beads remain recognisable as discrete particles, although surface roughening and partial fragmentation are observed after extended cycling. These morphological changes are consistent with the aggressive nature of the thermal cycling protocol and the repeated swelling–contraction of the hydrogel matrix. Together with the post-cycling DVS profiles, the results indicate that the beads retain water-sorption functionality after severe accelerated ageing, although the changes in isotherm shape and morphology suggest partial restructuring of the composite architecture. Because this test imposes harsher conditions than typical day–night AWH operation, the results support the potential of the materials for cyclic use, while further device-level validation under realistic airflow, irradiation, and humidity profiles remains necessary.

### 2.6. Comparative Performance Assessment with Reported AWH Sorbents

To contextualise the performance and practical relevance of the MOF/SA/GO composite beads, Table 1 compares the present materials with selected MOF-based composites, alginate hydrogels, and salt-assisted sorbents reported for atmospheric water harvesting. Direct comparison should be approached cautiously because the reported materials differ in composition, MOF-to-matrix ratio, drying method, sorption mechanism, testing duration, humidity range, and normalisation basis. In particular, the very high capacities reported for salt-assisted and highly swellable hydrogel systems may include substantial contributions from salt hydration, deliquescence, hydrogel swelling, and capillary water retention, rather than exclusively adsorption within permanent porosity.

GO–MIL-101(Cr)/alginate beads have already been reported [27]. The present study extends this materials approach through a comparison of MIL-101(Cr)- and MIL-100(Fe)-containing beads, including corresponding GO-free controls, and evaluates their water-vapour sorption, fixed-time solar water release, and cycling behaviour.

In the present work, incorporation of MIL-100(Fe) increased the water uptake of the alginate matrix from 0.32 to 0.38 g·g^−1^, corresponding to an improvement of approximately 19%, whereas MIL-101(Cr) produced the highest uptake of 0.48 g·g^−1^, corresponding to an improvement of approximately 50%. By contrast, incorporation of GO alone decreased the equilibrium uptake to 0.25 g·g^−1^, and its addition to the MOF-containing beads did not further increase the equilibrium adsorption capacity. These results indicate that in the present bead formulation, enhanced water uptake originates primarily from the porous MOF phase, while the main role of GO is associated with photothermal heat generation rather than increased equilibrium sorption.

The maximum capacities of the prepared beads remain below those reported for several salt-containing or highly swellable sorbents under moderate-to-high humidity. Nevertheless, the present materials display highly reversible sorption behaviour during isothermal DVS measurements. Desorption at 30 °C upon decreasing RH to 5% resulted in desorption-to-adsorption capacity ratios of approximately 92% for SA, 97% for SA/GO, 98% for Fe/SA, 98% for Fe/SA/GO, 97% for Cr/SA, and 98% for Cr/SA/GO. This indicates that a substantial fraction of the adsorbed water can be removed by humidity swing alone, without increasing the sample temperature. Such behaviour is relevant to arid and semi-arid environments, where low ambient humidity may assist passive or low-energy regeneration.

Low-temperature regeneration was further evaluated through photothermal desorption experiments. After 90 min under 1-sun irradiation, the fractions of initially adsorbed water released were 51.5%, 65.3%, and 69.8% for SA, Fe/SA, and Cr/SA, respectively. The corresponding GO-containing formulations released 62.8%, 73.4%, and 75.7%, respectively. These results confirm that GO improves solar-energy conversion and promotes water release under mild irradiation conditions.

Overall, the proposed beads should be regarded as a compromise between water capacity, reversibility, photothermal responsiveness, and processability. Although they do not outperform the highest-capacity salt-assisted or highly swellable hydrogel sorbents on a gravimetric basis, the combination of MIL porosity, Ca^2+^-crosslinked alginate structuring, and GO-assisted photothermal regeneration provides a mechanically manageable route toward solar-assisted AWH sorbents, without additional hygroscopic-salt impregnation. It should be emphasised that the present study is a materials-level evaluation of structured sorbent beads and does not include device-level liquid-water collection, airflow optimisation, condenser integration, or outdoor AWH validation.

## 3. Conclusions

This study demonstrates the design of structured photothermal sorbents based on MOF–alginate composite beads for solar-driven atmospheric water harvesting. By embedding MIL-101(Cr) and MIL-100(Fe) within Ca^2+^-crosslinked sodium alginate and incorporating graphene oxide as a photothermal component, the resulting materials integrate water adsorption and solar-assisted water release in a scalable structured bead architecture.

Dynamic vapour sorption measurements show formulation-dependent water uptake, with the Cr-containing beads reaching higher high-RH loadings than the Fe-containing beads, while incorporation into the alginate matrix modifies the sorption profiles of the parent MOFs. The alginate matrix contributes additional hydrophilic adsorption sites while providing a mechanically stable and permeable framework for vapour transport. Time-resolved DVS measurements further showed that Cr/SA/GO combines its higher water uptake with faster overall water exchange than Fe/SA/GO, particularly at high relative humidity, consistent with the larger accessible pore volume and pore apertures of MIL-101(Cr). Photothermal experiments under simulated sunlight reveal that GO enhances solar heating and improves the fraction of released water for all bead formulations.

After more than 2000 accelerated adsorption–desorption cycles, the MOF-containing beads retain water-sorption functionality, with approximately 60% of their initial uptake at 90% RH preserved, while SA/GO maintains an essentially unchanged uptake. These results support the resilience of the Ca^2+^-crosslinked hydrogel bead architecture under severe ageing conditions, although post-cycling structural and leaching analyses would be required to confirm full preservation of the embedded MOF phases. Because these tests impose harsher conditions than typical day–night AWH operation, the results suggest good potential for sustained cyclic operation, although device-level validation under realistic airflow, irradiation, and humidity profiles remains necessary.

Overall, the combination of MOF adsorption capacity, GO-enabled photothermal regeneration, and scalable alginate bead structuring represents a promising strategy for solar-assisted atmospheric water harvesting. Future work should focus on device-level testing under realistic airflow, irradiation, and day–night humidity/temperature profiles to quantify long-term water productivity.

## 4. Materials and Methods

### 4.1. Materials

Iron(II) sulphate (FeSO_4_·7H_2_O, ≥98%), chromium nitrate (Cr(NO_3_)_3_·9H_2_O), 1,4-benzenedicarboxylic acid (H_2_BDC, 98%), and 1,3,5-benzenetricarboxylic acid (H_3_BTC, 98%) were purchased from Thermo Scientific (Waltham, MA, USA). Calcium chloride (≥97%), sodium alginate (technical grade), anhydrous DMF (99.8%), and sodium hydroxide (NaOH, ≥98%) were purchased from Sigma-Aldrich (St. Louis, MO, USA). Graphene oxide powder was purchased from Graphenea (San Sebastián, Spain). RPE water for synthesis and analysis was acquired from Carlo Erba (Cornaredo, Italy).

### 4.2. Synthesis of Pristine MOF Powders

#### 4.2.1. Synthesis of MIL-101(Cr)

MIL-101(Cr) was obtained by dissolving Cr(NO_3_)_3_·9H_2_O (13.02 mmol) and H_2_BDC (13.02 mmol) with a molar ratio of 1:1 in 50 mL of Milli-Q water. The mixture was stirred at room temperature (25 ± 2 °C) for 1 h and sonicated for 10 min, then heated up to 220 °C and kept for 8 h in a Teflon-lined stainless-steel autoclave. The product was then washed with Milli-Q water to remove unreacted material and dried at 80 °C for 4 h. The dry material was ground to a fine dark green powder.

#### 4.2.2. Synthesis of MIL-100(Fe)

MOF MIL-100(Fe) was prepared following an upscaled variation of the method reported by Tan and Foo [32]. In detail, 5.72 g (27.2 mmol) of H_3_BTC was stirred vigorously to be dissolved in 90 mL of aqueous NaOH (1.0 M). The H_3_BTC solution was added dropwise to an iron(II) salt solution, which was prepared beforehand by dissolving 11.45 g (41.2 mmol) of FeSO_4_·7H_2_O in 356 mL of RPE water. The solution mixture was stirred for 24 h to allow for crystallisation at ambient temperature. The crystallised powder was collected via filtration and washed sequentially with water and ethanol for the neutralisation of solution pH and removal of unreacted material. The pH was checked after the complete addition of all the reactants and multiple times during the washing procedure. The final product was dried at 80 °C for 4 h.

### 4.3. Fabrication of MOF–Alginate Composite Beads

Suspension A (MOF/GO dispersion). Either MIL-101(Cr) or MIL-100(Fe) powder (0.50 g) was dispersed together with graphene oxide (GO, 10 wt.% relative to MOF, i.e., 0.05 g) in deionised water (5.0 mL). The slurry was stirred magnetically at ambient temperature until visually homogeneous (typically 10–15 min). When necessary, brief bath sonication (≤5 min) was used to break soft agglomerates without appreciable heating.

Solution B (sodium alginate). Sodium alginate (0.50 g) was dissolved in deionised water (10.0 mL) at 40 °C under magnetic stirring until a clear, bubble-free solution was obtained.

Suspension A was added dropwise to Solution B over ~2 min under continuous stirring (≈400 rpm). The mixture (total solids ≈ 1.05 g in 15 mL) was maintained at 40 °C under gentle agitation to promote uniform MOF/GO distribution within the alginate matrix. The composite slurry was extruded through a syringe (21–23 G needle) into a gently stirred CaCl_2_ bath (0.1 M) from a height of ~5 cm to form spherical beads. Beads were cured for 12 h, then collected by sieving, and washed three times with deionised water to remove excess Ca^2+^. Beads were dried at 80 °C overnight.

For clarity, the sample nomenclature used throughout the manuscript is as follows: beads containing MIL-101(Cr) and MIL-100(Fe) are denoted as Cr/SA and Fe/SA, respectively, while the corresponding GO-containing composites are denoted as Cr/SA/GO and Fe/SA/GO. Beads prepared without MOF are labelled as SA and SA/GO, depending on the absence or presence of GO.

### 4.4. Characterisation Methods

For the studied materials, powder X-ray diffraction (PXRD) patterns were obtained on a Bruker D8 ADVANCE (Billerica, MA, USA), with a scanning range of 2–50°, at a scanning rate of 0.05°/s. A scanning electron microscope (SEM-FEI Quanta FEG 450, Hillsboro, OR, USA) was used to characterise the particle morphology and crystal size. Specific surface area was quantified using the Brunauer–Emmett–Teller (BET) method by a QUANTACHROME NOVA 1200e (Boynton Beach, FL, USA), while pore volume was assessed using the Barrett–Joyner–Halenda (BJH) approach from the desorption isotherms. Fourier-transform infrared (FTIR) spectra were recorded on a PerkinElmer Spectrum Two spectrometer (PerkinElmer Inc., Waltham, MA, USA) using attenuated total reflectance (ATR) mode in the 4000–500 cm^−1^ range.

### 4.5. Water-Vapour Sorption Measurements

The water adsorption properties of all bead formulations were studied by dynamic vapour sorption (DVS) analysis, with mass changes (water uptake) recorded during controlled hydration–dehydration cycles. Isotherms were measured at 30 °C across a relative humidity (RH) range of 5–90% by means of ProUmid SPS23 gravimetric DVS Analyser (ProUmid GmbH & Co. KG, Ulm, Germany). Relative humidity steps were set to 5% in the 5–40% RH range and 10% in the 40–90% RH range. Prior to analysis, samples were oven-dried at 80 °C overnight, followed by an in situ thermal stabilisation at 60 °C for 4 h and final equilibration at 30 °C and 5% RH. The DVS featured a microbalance with a precision of ±0.01 mg and a controller to regulate the vapour pressure within the measurement chamber. Time-resolved adsorption–desorption measurements were additionally performed on Cr/SA/GO and Fe/SA/GO beads at 30 °C. Separate adsorption steps were conducted at 10, 40, and 70% RH, followed by desorption under dry conditions at 80 °C. Mass changes were continuously recorded as a function of time and expressed relative to the dry mass of the corresponding sample.

### 4.6. Solar-Driven Desorption and Photothermal Measurements

Photothermal heating-triggered water desorption was performed on a solar simulator (LOT-Oriel model LS0100-1000, Darmstadt, Germany) equipped with a xenon lamp at ambient conditions (25 °C and 50% RH), under 1-sun (AM 1.5G). Water desorption was quantified from the sample mass loss after 90 min of continuous irradiation. Prior to solar desorption experiments, all samples were equilibrated at 70% RH for 18 h to establish a reproducible hydrated state. The surface temperature evolution of the beads during irradiation was monitored using a FLIR (model FLIR-E63900, FLIR Systems AB, Täby, Sweden) infrared thermal camera. Representative thermographic images were displayed using the FLIR thermal blending mode, which combines infrared and visible-light information to facilitate localisation of the heated sample region. The displayed temperature range was automatically adjusted by the acquisition software; therefore, the thermographic images were used for qualitative visualisation, whereas quantitative comparisons were based on the measured surface-temperature values. In particular, surface-temperature values were obtained from the thermographic region corresponding to the beads.

The residual water content during desorption was calculated as

(2) $$Residual\;water\;\left(\%\right)=\frac{m_{t}-m_{0}}{m_{b}}\times 100$$

where $m_{t}$ is the mass of the beads at time t during evaporation, $m_{0}$ is the mass of the fully dried beads, and $m_{b}$ is the mass of water initially adsorbed after equilibration at 70% RH. The released-water fraction (%) was calculated as 100 minus the residual-water percentage after 90 min of irradiation.

### 4.7. Cycling and Durability Testing

#### 4.7.1. Short-Term Wetting–Drying Repeatability Test

Short-term wetting–drying repeatability was evaluated gravimetrically over ten consecutive cycles for the Fe/SA, Fe/SA/GO, Cr/SA, and Cr/SA/GO bead formulations. For each cycle, the wetting step was performed in an environmental chamber at 35 °C and 90% RH for 2 h. After wetting, the samples were weighed immediately to determine the hydrated mass. Regeneration was then carried out in a laboratory oven at 80 °C for 12 h, after which the samples were cooled briefly under ambient laboratory conditions and weighed again to determine the post-drying mass.

All masses were recorded using the same sample holders throughout the experiment. The tare mass of each empty holder was measured separately and subtracted during data processing so that the water uptake was normalised to the dry bead mass only.

#### 4.7.2. Accelerated Ageing Stress Test

The durability of the composite materials was tested in a custom-designed experimental apparatus engineered for accelerated ageing under repeated adsorption–desorption conditions, described in Ref. [33]. This system consists primarily of a vacuum chamber housing a heat exchanger (HEX) plate. The bead samples were positioned on the heat-exchanger plate and secured using an aluminium mould to ensure reproducible thermal contact during repeated adsorption–desorption cycling. The adsorption temperature was set to 30 °C and the desorption temperature to 80 °C. The thermostatic bath connected to the evaporator was set to 20 °C. Regarding the cycle duration, the automated valves were configured for a 10-min adsorption phase and a 7-min desorption phase. The adsorption phase was made longer than desorption to accommodate the extended cooling time during water vapour adsorption. For durability testing, the composites underwent more than 2000 accelerated adsorption–desorption cycles to assess physical integrity and water sorption properties.

## Figures and Tables

**Figure 1:**
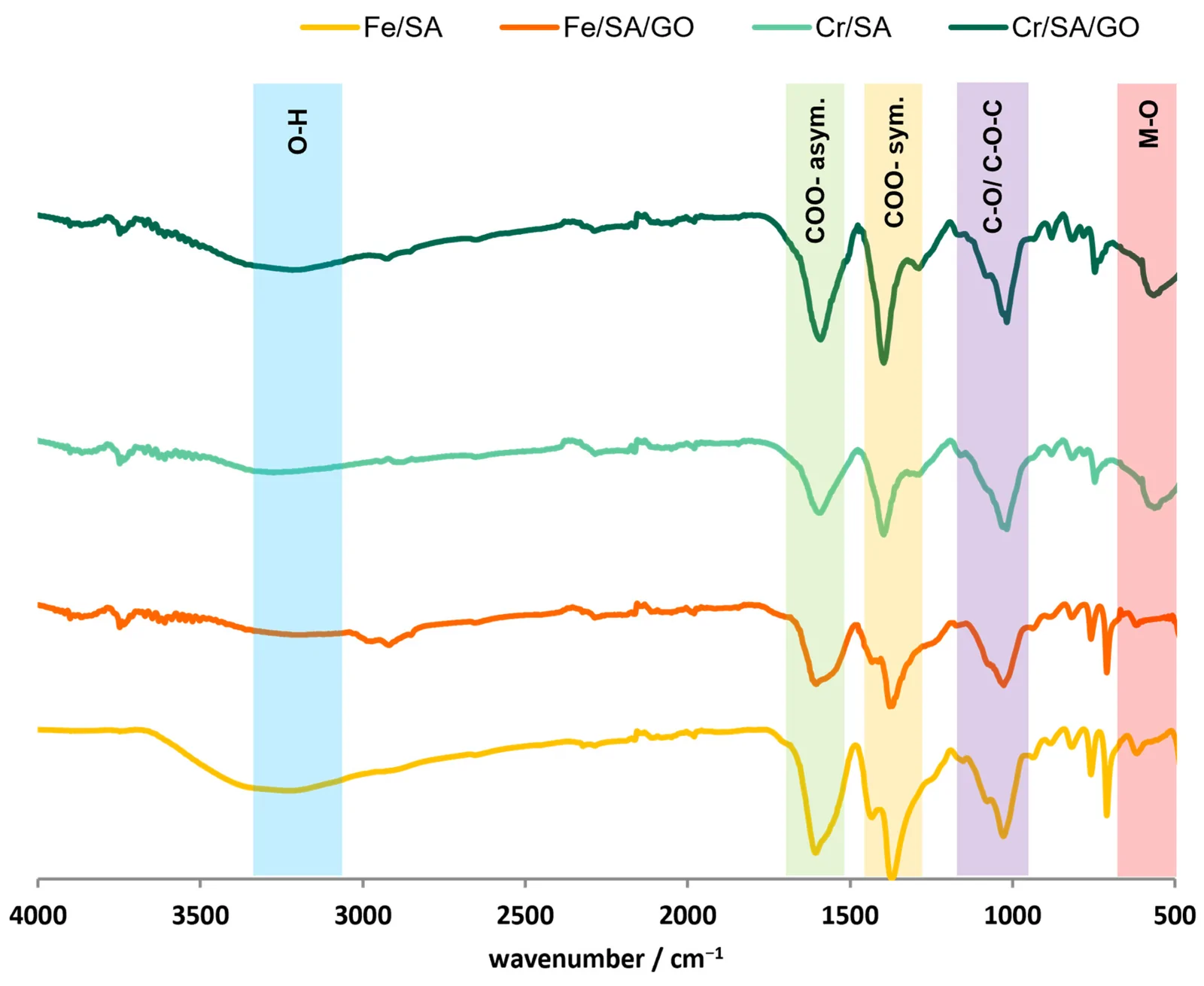
FTIR transmittance spectra of MOF–alginate composite beads.

**Figure 2:**
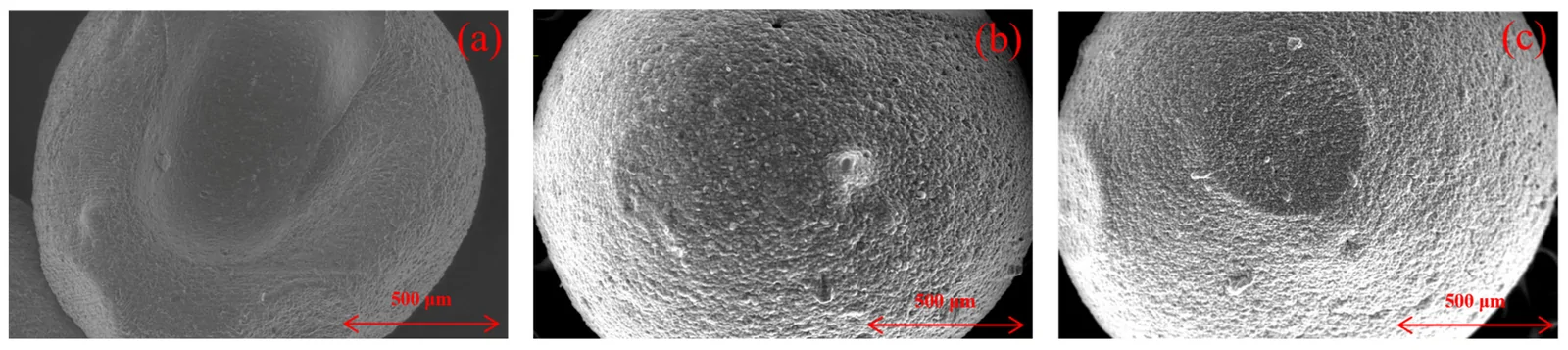
Scanning electron microscopy (SEM) images of the composite beads. Representative SEM micrographs showing the morphology of (**a**) SA/GO beads, (**b**) Cr/SA/GO beads, and (**c**) Fe/SA/GO beads.

**Figure 3:**
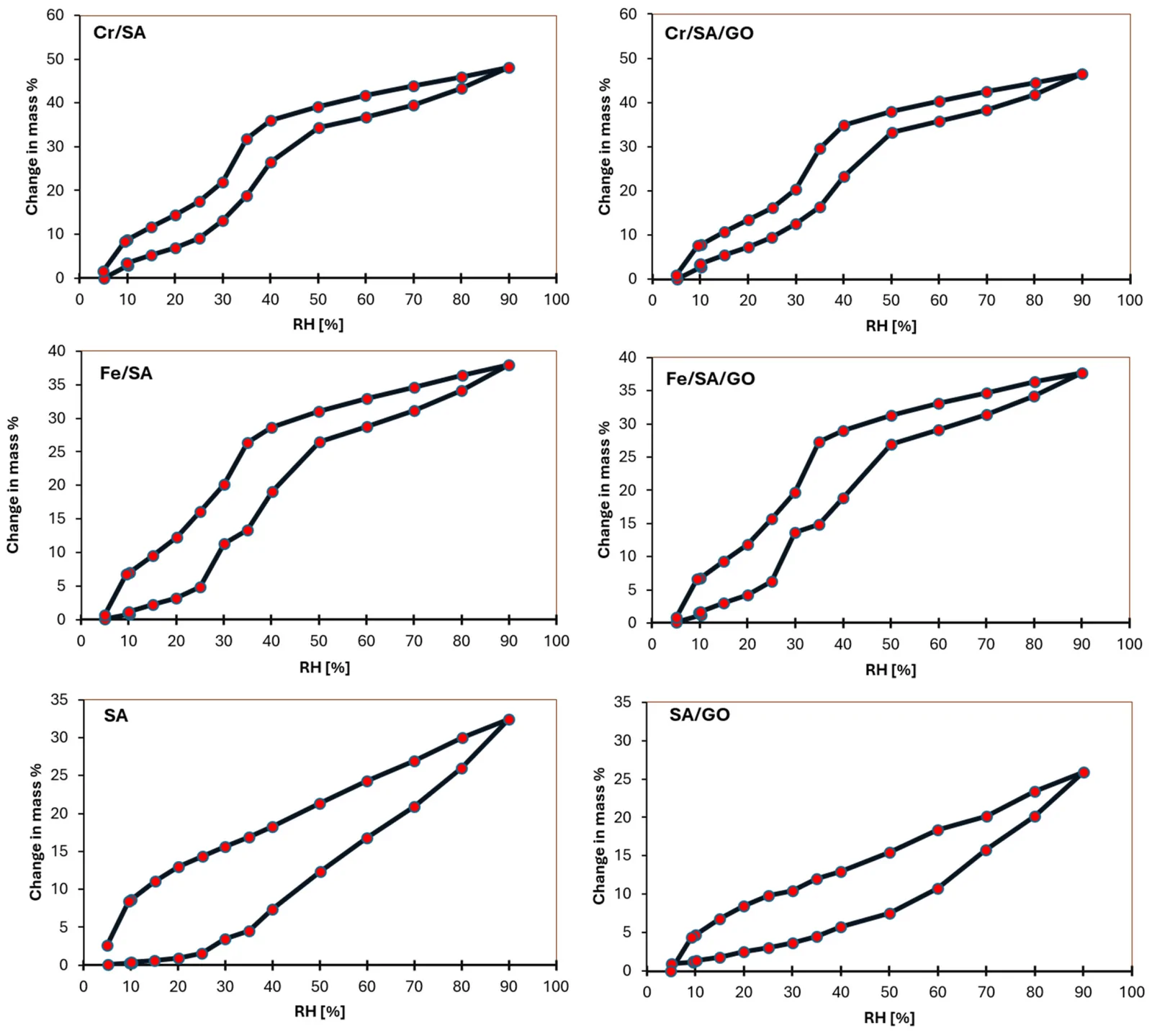
Water vapour adsorption–desorption isotherms of the composite beads measured by DVS at 30 °C. Isotherms for all the beads were measured over the 5–90% RH range. The lower and upper branches correspond to adsorption and desorption, respectively.

**Figure 4:**
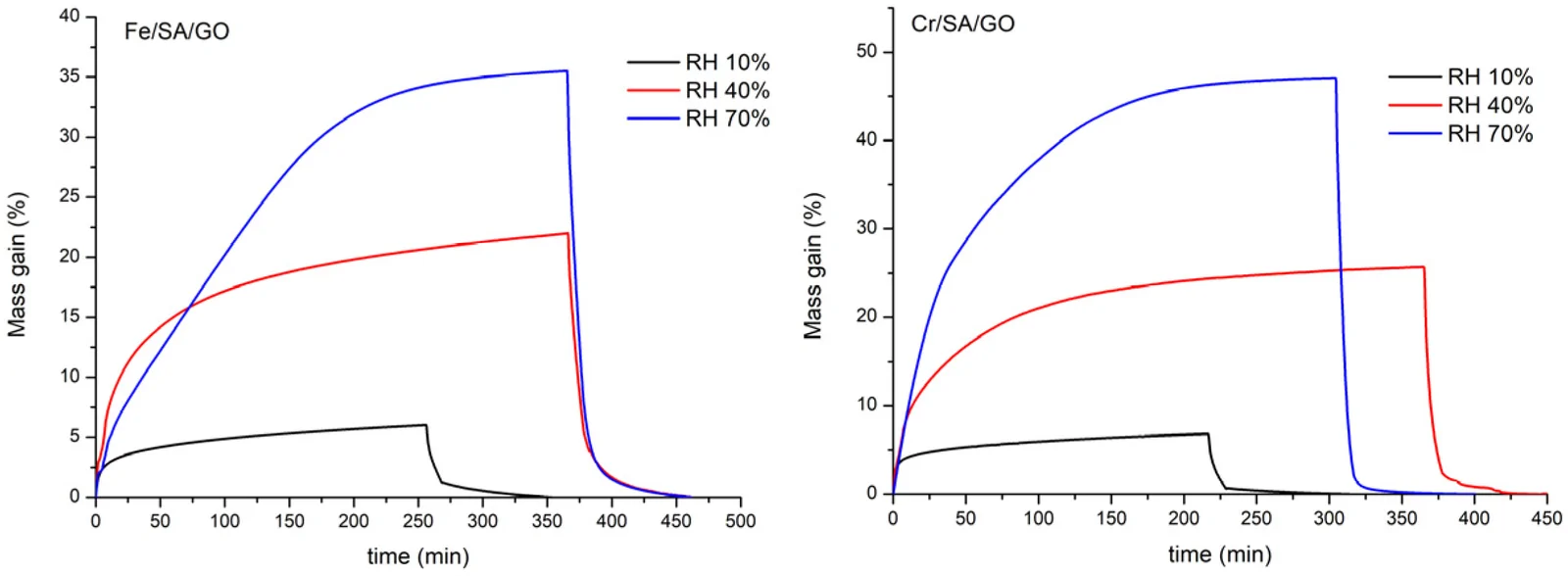
Time-resolved water-vapour adsorption–desorption profiles of (**left**) Fe/SA/GO and (**right**) Cr/SA/GO beads at 30 °C under 10, 40, and 70% RH, followed by desorption under dry conditions at 80 °C.

**Figure 5:**
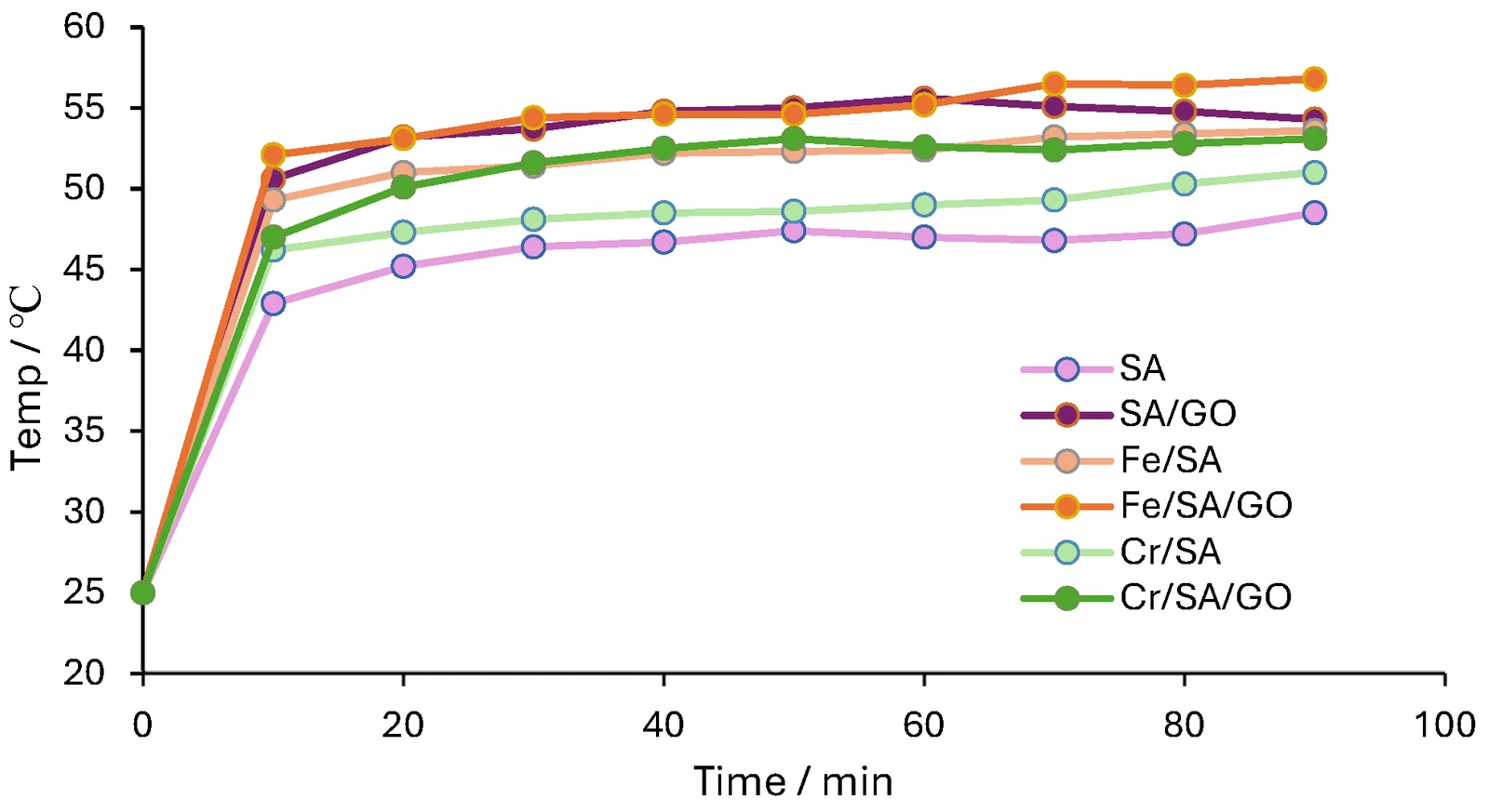
Photothermal heating behaviour of the composite beads under simulated solar irradiation (1 sun, AM 1.5G). Surface temperature evolution of SA, SA/GO, Fe/SA, Fe/SA/GO, Cr/SA, and Cr/SA/GO beads monitored by infrared thermography during illumination.

**Figure 6:**
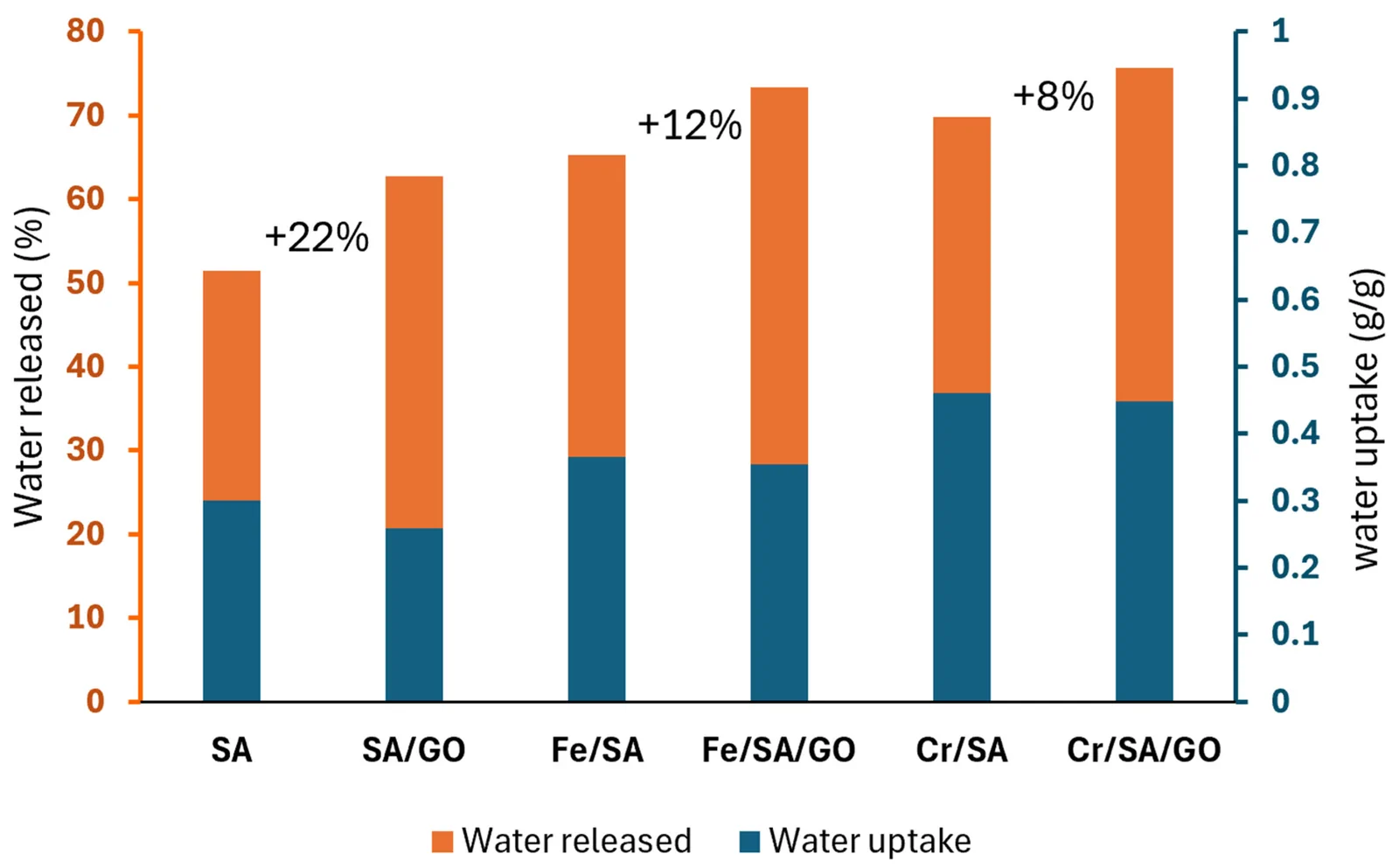
Solar-driven water desorption performance of the composite beads. ((**left**) Y axis, orange bars) Fraction of water released after 90 min of simulated solar irradiation (1 sun) for SA, SA/GO, Cr/SA, Cr/SA/GO, Fe/SA, and Fe/SA/GO beads. Percentage increases are calculated relative to the released-water fraction of the corresponding GO-free formulation. ((**right**) Y axis, blue bars) Corresponding equilibrium water uptake of the samples.

**Figure 7:**
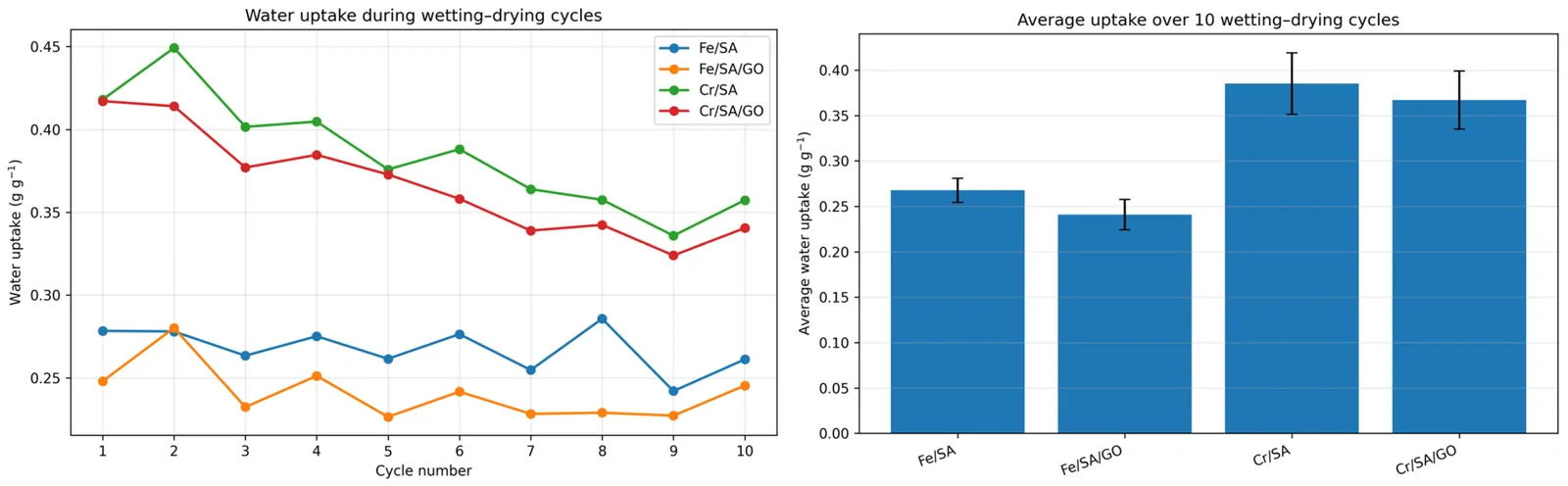
Wetting–drying cycling stability of MOF/alginate composite beads. (**left**) Water uptake per cycle, calculated as $q=(m_{\mathrm{wet}}-m_{\mathrm{dry}})/(m_{\mathrm{dry}}-m_{\mathrm{tare}})$, for Fe/SA, Fe/SA/GO, Cr/SA, and Cr/SA/GO beads over ten consecutive cycles. Wetting was performed at 35 °C and 90% RH, while drying was conducted at 80 °C. (**right**) Mean water uptake across ten consecutive cycles; error bars represent the standard deviation across cycles.

**Figure 8:**
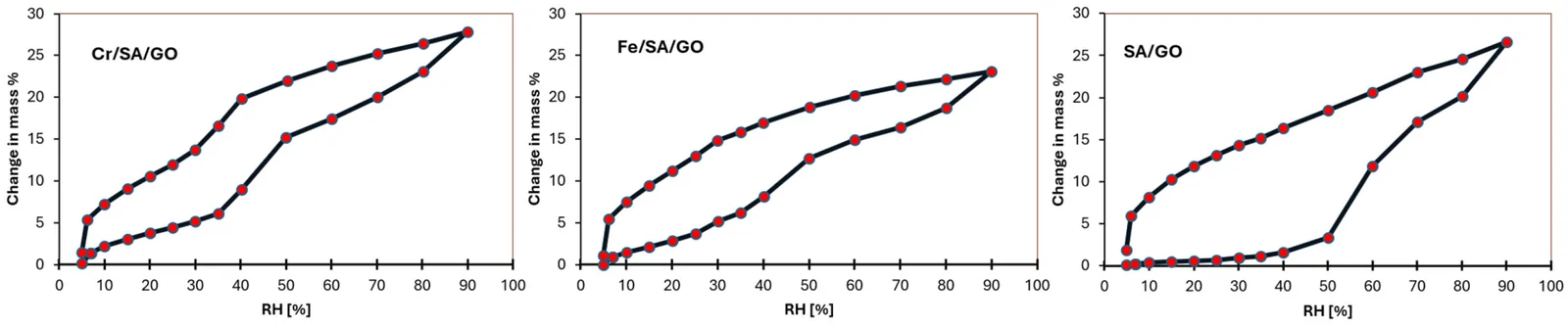
Water vapour adsorption–desorption isotherms of MOF/SA/GO beads measured after more than 2000 accelerated adsorption–desorption cycles (adsorption at 30 °C; desorption at 80 °C).

**Figure 9:**
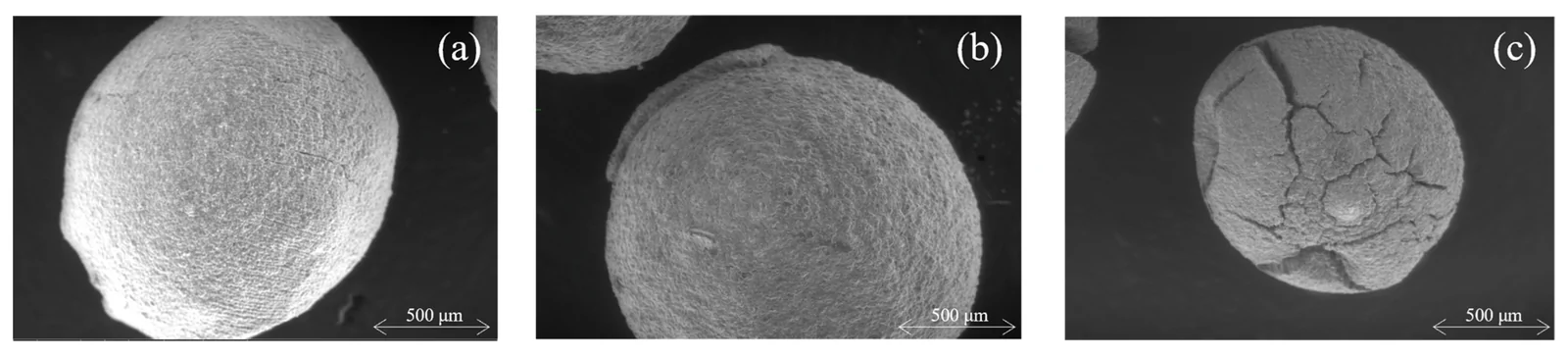
SEM images of composite beads after accelerated stress cycling. Post-cycling morphology of (**a**) Fe/SA/GO, (**b**) Cr/SA/GO, and (**c**) SA/GO beads after >2000 adsorption–desorption cycles.

**Table 1:** Comparative assessment of the adsorption and regeneration of the MOF/SA/GO beads with selected AWH sorbents reported in the literature.

Material/System	Sorbent Type	Method/Basis	Adsorption Condition	Water Uptake, (q_ads_)/g·g^−1 a^	Regeneration/Release Condition	Released or Recoverable Water ^b^	Ref.
SA	Ca^2+^-crosslinked alginate bead	DVS humidity swing	30 °C, 90% RH	0.323	30 °C, 90 → 5% RH	0.298 g·g^−1^ ^c^	This work
SA/GO	Alginate/GO bead	DVS + solar screening	30 °C, 90% RH	0.25	1 sun, 90 min	+22% in solar-assisted released-water fraction vs. SA ^d^	This work
Fe/SA/GO	MIL-100(Fe)/alginate/GO bead	DVS + solar screening	30 °C, 90% RH	0.376	30 °C, 90 → 5% RH; 1 sun, 90 min	0.369 g·g^−1^ ^c^; up to ~75% solar release ^d^	This work
Cr/SA/GO	MIL-101(Cr)/alginate/GO bead	DVS + solar screening	30 °C, 90% RH	0.465	30 °C, 90 → 5% RH; 1 sun, 90 min	0.456 g·g^−1^ ^c^; up to ~75% solar release ^d^	This work
GO–MIL-101(Cr)/alginate beads	MOF–alginate–GO hydrogel bead	Fixed-RH gravimetric chamber	25 °C, 90% RH, 12 h	3.14 ^e^	Thermal, 60–80 °C	91% at 30 min at 60 °C; 100% at 30 min at 80 °C ^e^	[27]
MOF-808/CaCl_2_/Fe_3_O_4_/alginate beads	Salt-assisted magnetic MOF/alginate bead	Gravimetric AWH + induction heating	25 °C, 75% RH, 24 h	1.02 ^e^	Magnetic induction, 171 A	~85% release in 30 min; full release in ~100 min ^e^	[17]
Alginate–graphite hydrogel beads	Low-salt Ca-alginate/graphite hydrogel	Gravimetric kinetics + modelling	21 °C, RH_avg_ ~37–38%	0.60–0.65 ^e^	Thermal, 60–100 °C	0.52–0.53 g·g^−1^ after kinetic test; predicted 1.6–2.9 L kg^−1^ day^−1^ ^e^	[28]
Loofah/Ca-alginate/CaCl_2_/carbon ink, ILCA	Salt-assisted photothermal scaffold	STA + water-collection test	30 °C, 80% RH	up to ~2.4 ^e^	80 °C STA; 1 sun solar release	~2.2 g·g^−1^ in STA; 45.0% collected/adsorbed water in lab device ^e^	[29]
CMC–CaCl_2_ hydrogel beads	Salt-loaded biodegradable hydrogel	Fixed-RH gravimetry + VTI cycling	25 °C, 90% RH	4.81 ^e^	Drying at 100 °C	Stable over 3 adsorption–desorption cycles; 1.79–4.81 g·g^−1^ over 30–90% RH ^e^	[30]
Semi-IPN PPy/salt/GO/alginate sorbent	Salt-assisted photothermal hydrogel	Indoor AWH + solar release	23 °C, 90% RH	up to 7.15 ^e^	1 sun; surface temperature up to ~71 °C	74% water-collection efficiency; 3.36 L kg^−1^ per cycle ^e^	[15]
PNIPAM/Ca-alginate thermoresponsive gel	Thermoresponsive hydrogel	Moisture absorption + temperature-triggered water release	27 °C, 80% RH	~0.6 ^e^	Heating to 50 °C	~0.12 g·g^−1^ liquid water oozed ^e^	[31]

^a^ Water uptake refers to the total gravimetric mass increase under the reported humidity conditions. Depending on the material, this value may include MOF adsorption, hydrogel hydration/swelling, salt hydration, deliquescence, capillary condensation, or water retained in meso/macropores. ^b^ Released or recoverable water is reported as given in the corresponding study and may refer to humidity-swing desorption, thermal desorption, solar release, magnetic-induction release, STA-derived mass loss, VTI analysis, or device-level collected water. ^c^ For this work, recoverable water from DVS refers to the amount released during the humidity-swing desorption branch from 90% to 5% RH at 30 °C. ^d^ For this work, solar release refers to the fraction of initially adsorbed water released after 90 min under 1-sun irradiation after equilibration at 70% RH for 18 h. ^e^ Literature values are reported as given in the original studies and are not always directly comparable because they may derive from different testing protocols, including DVS, fixed-RH gravimetry, thermal regeneration, solar regeneration, magnetic induction, STA measurements, VTI analysis, or device-level water collection. Abbreviations: SA, sodium alginate; GO, graphene oxide; MOF, metal–organic framework; MIL, Materials Institute Lavoisier framework; DVS, dynamic vapour sorption; RH, relative humidity; STA, simultaneous thermal analysis; VTI, vapour sorption analyser; CMC, carboxymethyl cellulose; PPy, polypyrrole; IPN, interpenetrating polymer network; ILCA, loofah-grafted calcium alginate with carbon ink; Fe_3_O_4_, iron oxide; CaCl_2_, calcium chloride.

## Data Availability

The raw data supporting the conclusions of this article will be made available by the authors on request.

## Supplementary Materials

The following supporting information can be downloaded at: https://www.mdpi.com/article/10.3390/gels12100869/s1, Figure S1: PXRD patterns of pristine MIL-101(Cr), fresh Cr/SA/GO beads and Cr/SA/GO beads after ten wetting–drying cycles (left), and the corresponding MIL-100(Fe), Fe/SA/GO and cycled Fe/SA/GO samples (right); Figure S2: SEM images of MIL-100(Fe) (left) and MIL-101(Cr) (right); Figure S3: SEM–EDS elemental mapping of Fe/SA/GO and Cr/SA/GO composite beads. Representative SEM images and corresponding elemental maps showing the distribution of Ca and Fe in Fe/SA/GO, and Ca and Cr in Cr/SA/GO; Figure S4: Cross-sectional SEM images of Fe/SA/GO (left) and Cr/SA/GO (right) composite beads; Figure S5: FTIR spectra of the reference powders; Figure S6: Water vapor adsorption–desorption isotherms of the pristine powders measured by DVS at 30 °C. Isotherms for both materials were measured over the 0–70% RH range; Figure S7: Representative FLIR thermographic images of SA, Fe/SA, Cr/SA, SA/GO, Fe/SA/GO and Cr/SA/GO beads after 90 min of simulated 1-sun irradiation. Images were acquired using Thermal Blending, combining infrared and visible-light information to facilitate localisation of the heated bead region. The thermograms are provided as qualitative visual support for the surface-temperature measurements reported in the main text. Because the displayed temperature range was automatically adjusted for each acquisition, colour differences between individual panels should not be interpreted quantitatively; Table S1: Textural properties of powders from N_2_ sorption at −196 °C.
