# Supplementary material for: Photothermal MOF–Alginate Hydrogel Beads for Solar-Driven Atmospheric Water Harvesting
Source: Gels. 2026 Sep 25;12(10):869. doi: 10.3390/gels12100869 (PMC13704661; doi:10.3390/gels12100869)
Supplement: Supplementary file 1 [file gels-12-00869-s001.zip › gels-4480985-supplementary.pdf]

Supporting information

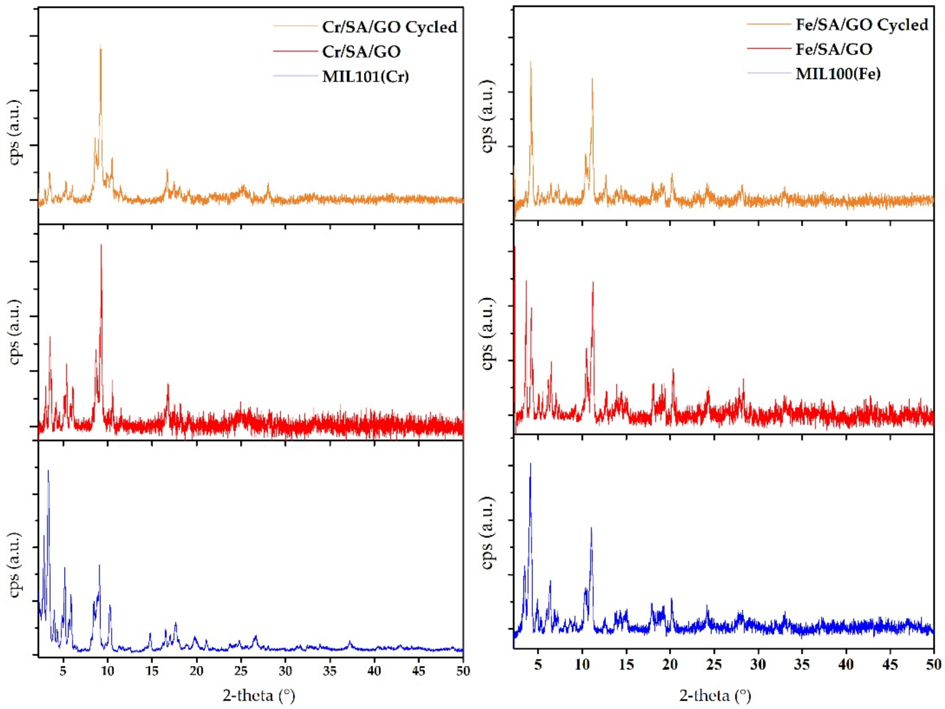

Figure S1: PXRD patterns of pristine MIL-101(Cr), fresh Cr/SA/GO beads and Cr/SA/GO beads after ten wetting–drying cycles (left), and the corresponding MIL-100(Fe), Fe/SA/GO and cycled Fe/SA/GO samples (right).

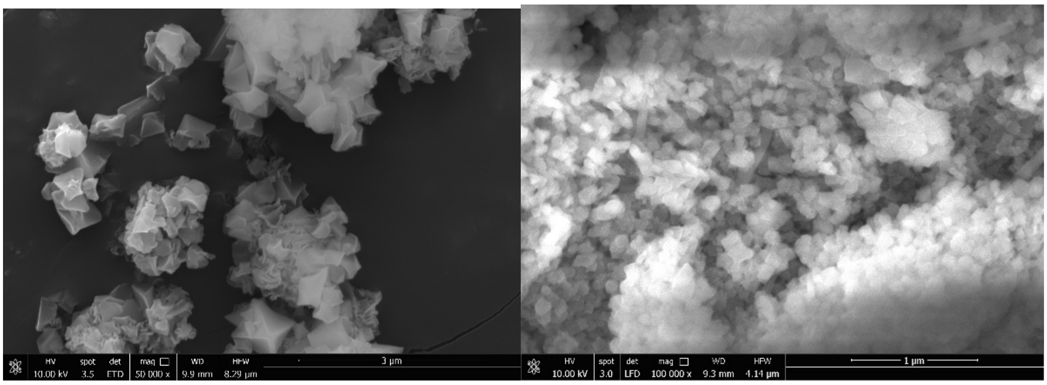

Figure S2: SEM images of MIL-100(Fe) (left) and MIL-101(Cr) (right).

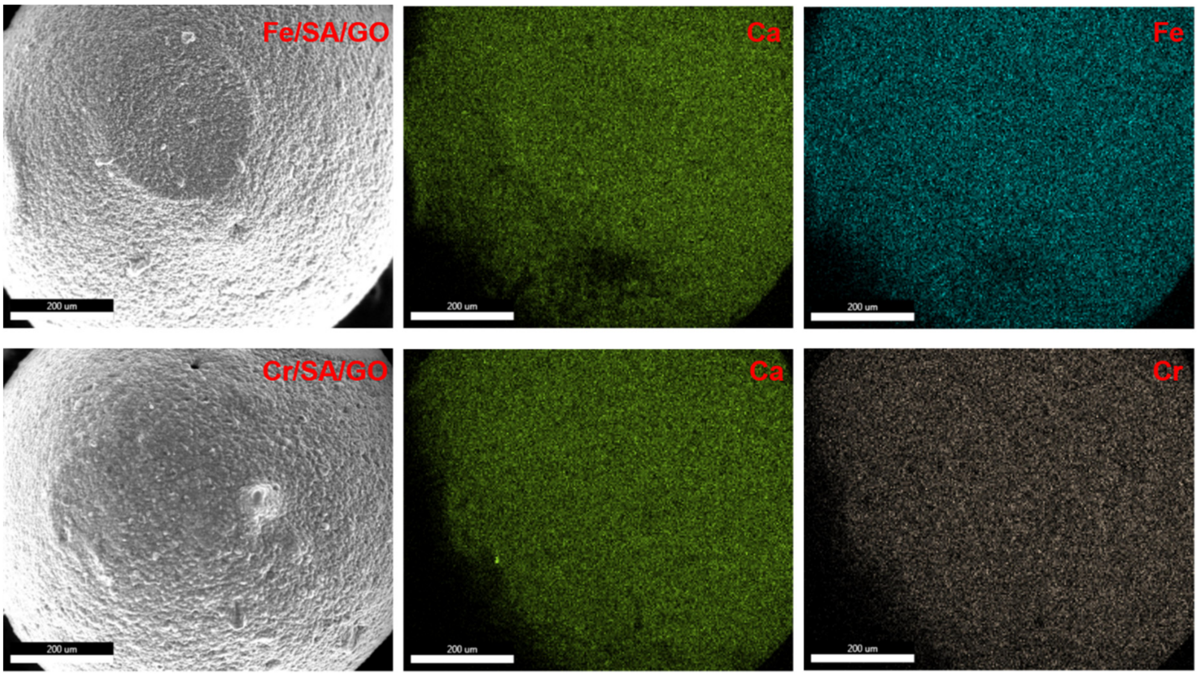

Figure S3. SEM–EDS elemental mapping of Fe/SA/GO and Cr/SA/GO composite beads. Representative SEM images and corresponding elemental maps showing the distribution of Ca and Fe in Fe/SA/GO, and Ca and Cr in Cr/SA/GO.

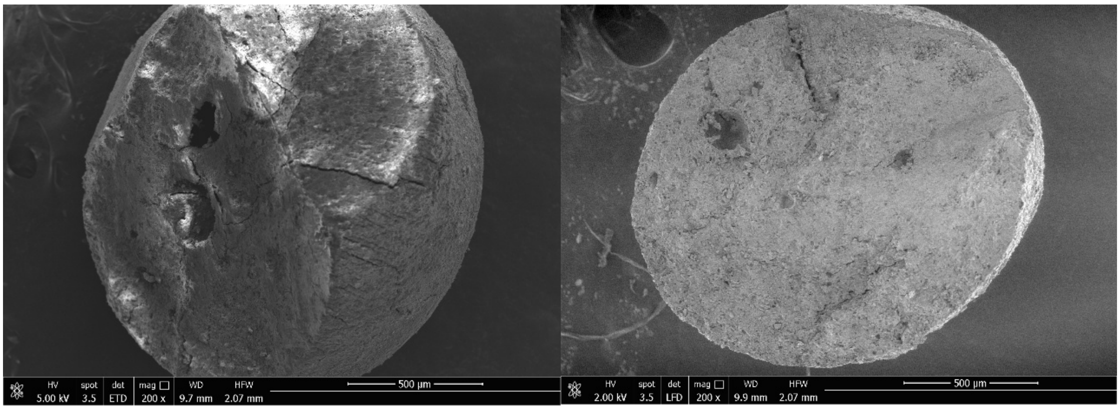

Figure S4. Cross-sectional SEM images of Fe/SA/GO (left) and Cr/SA/GO (right) composite beads.

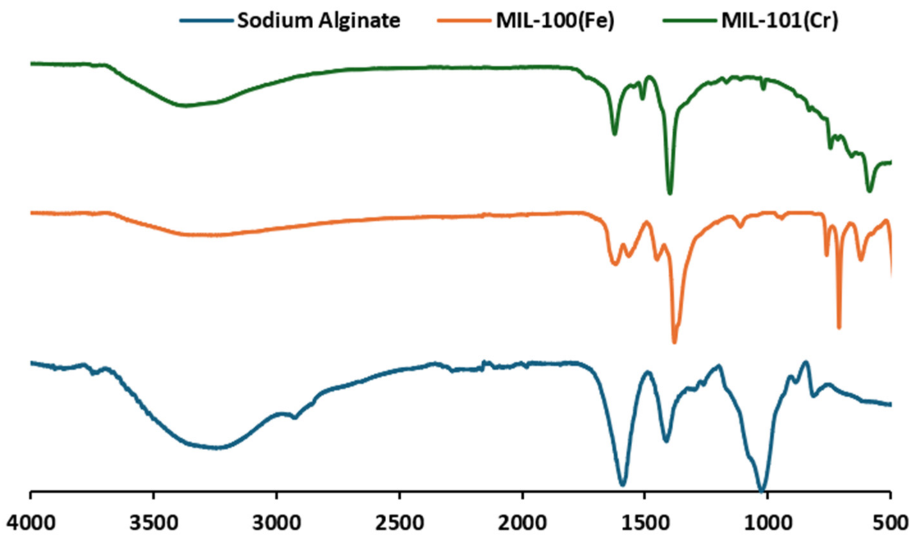

Figure S5. FTIR spectra of the reference powders.

Table S1. Textural properties of powders from N_2 sorption at -196 °C.

Sample	S_BET (m^2 g^{-1})	V_pore (cm^3 g^{-1})
MIL-100(Fe)	1449.26	0.69
MIL-101(Cr)	2796.96	1.82

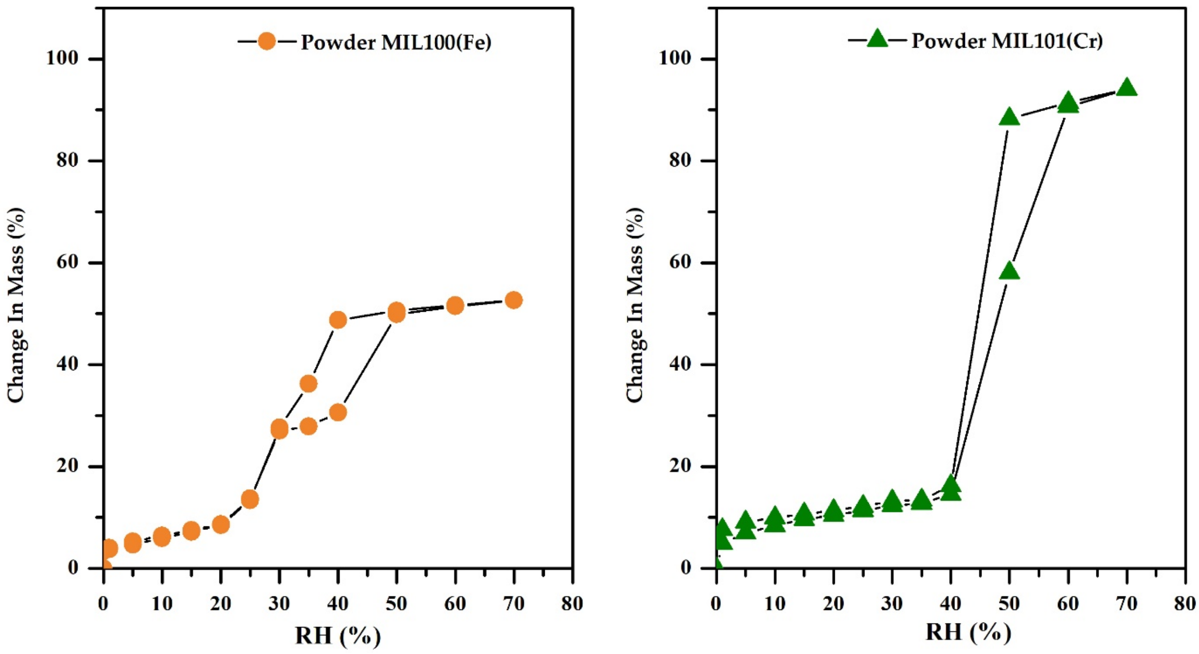

Figure S6. Water vapor adsorption–desorption isotherms of the pristine powders measured by DVS at 30 °C. Isotherms for both materials were measured over the 0–70% RH range.

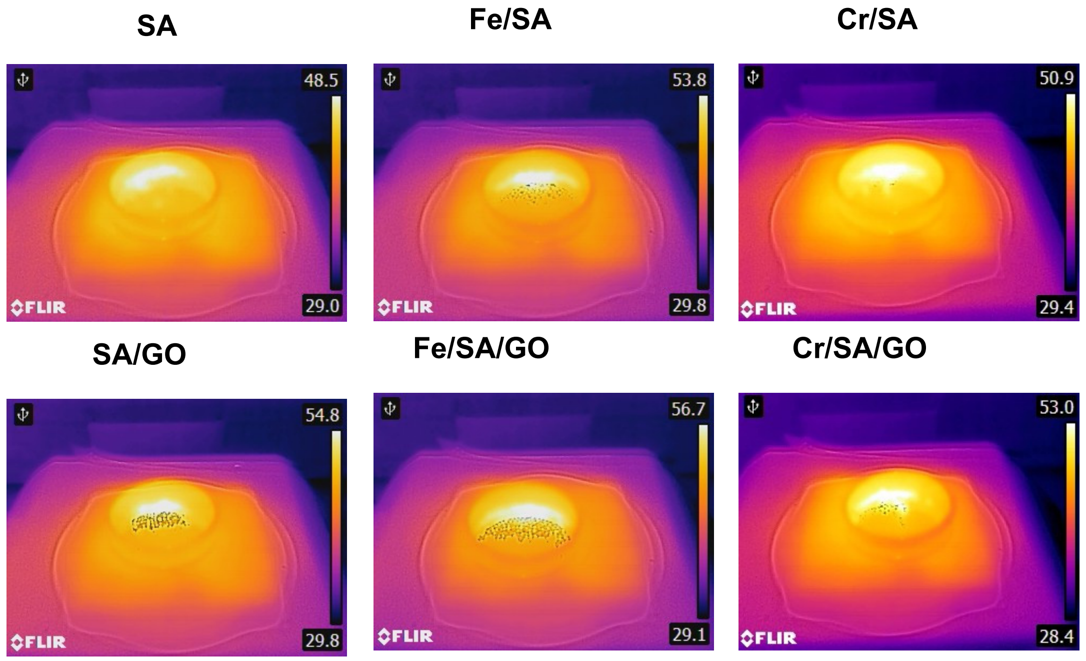

Figure S7. Representative FLIR thermographic images of SA, Fe/SA, Cr/SA, SA/GO, Fe/SA/GO and Cr/SA/GO beads after 90 min of simulated 1-sun irradiation. Images were acquired using Thermal Blending, combining infrared and visible-light information to facilitate localisation of the heated bead region. The thermograms are provided as qualitative visual support for the surface-temperature measurements reported in the main text. Because the displayed temperature range was automatically adjusted for each acquisition, colour differences between individual panels should not be interpreted quantitatively.
